# Highly-Stable Li_4_Ti_5_O_12_ Anodes Obtained by Atomic-Layer-Deposited Al_2_O_3_

**DOI:** 10.3390/ma11050803

**Published:** 2018-05-16

**Authors:** Jae Kook Yoon, Seunghoon Nam, Hyung Cheoul Shim, Kunwoo Park, Taeho Yoon, Hyung Sang Park, Seungmin Hyun

**Affiliations:** 1 Nano Mechanical Systems Research Division, Department of Nano Mechanics, Korea Institute of Machinery and Materials (KIMM), Daejeon 34103, Korea; jkyoon@kimm.re.kr (J.K.Y.); kwek14@kimm.re.kr (S.N.); scafos@kimm.re.kr (H.C.S.); 2 ISAC Research Inc., Daejeon 34036, Korea; kw.park@isacresearch.com (K.P.); th.yoon@isacresearch.com (T.Y.); hys.park@isacresearch.com (H.S.P.); 3 Department of Nanomechatronics, University of Science and Technology (UST), Daejeon 34113, Korea

**Keywords:** Li_4_Ti_5_O_12_, atomic-layer-deposited Al_2_O_3_, gas evolution

## Abstract

LTO (Li_4_Ti_5_O_12_) has been highlighted as anode material for next-generation lithium ion secondary batteries due to advantages such as a high rate capability, excellent cyclic performance, and safety. However, the generation of gases from undesired reactions between the electrode surface and the electrolyte has restricted the application of LTO as a negative electrode in Li-ion batteries in electric vehicles (EVs) and energy storage systems (ESS). As the generation of gases from LTO tends to be accelerated at high temperatures (40–60 °C), the thermal stability of LTO should be maintained during battery discharge, especially in EVs. To overcome these technical limitations, a thin layer of Al_2_O_3_ (~2 nm thickness) was deposited on the LTO electrode surface by atomic layer deposition (ALD), and an electrochemical charge-discharge cycle test was performed at 60 °C. The capacity retention after 500 cycles clearly shows that Al_2_O_3_-coated LTO outperforms the uncoated one, with a discharge capacity retention of ~98%. TEM and XPS analyses indicate that the surface reactions of Al_2_O_3_-coated LTO are suppressed, while uncoated LTO undergoes the (111) to (222) phase transformation, as previously reported in the literature.

## 1. Introduction

Lithium-ion batteries have been used as a power source for operating various small electronic devices, such as mobile phones and laptops. Moreover, mid- and large-size batteries are used in electronic vehicles (HEVs, PHEVs, and EVs) as well as in energy storage systems (ESS), which have recently been commercialized. Therefore, electrode materials with high capacity, high power, short charging times, and excellent lifetimes are essential for the performance and safety of the batteries meant for frequent use [1,2,3,4,5]. However, graphite and carbon-based materials, which are commercially used as negative electrodes, are now considered an impediment for fast-charging applications. One alternative to graphite is spinel Li_4_Ti_5_O_12_ (LTO), which exhibits an excellent rate capability with superior cycle-life performance compared to existing anode material candidates. Moreover, as LTO has a relatively high reaction potential (1.55 V vs. Li/Li^+^), an irreversible solid-electrolyte interphase (SEI) is not generated in the early stages of delithiation. Such advantageous characteristics highlight LTO as an appropriate material for electronic vehicles (HEVs, PHEVs, and EVs) and ESS [6,7,8,9,10,11,12,13,14,15,16,17,18,19,20,21,22,23].

One of the disadvantages of LTO is the degradation in the performance of the electrode caused by gas generation and electrolyte decomposition at the interface. As opposed to graphite and other Li-alloy anodes, the absence of an irreversible SEI layer causes LTO to be in direct contact with the electrolyte and hence to be vulnerable to side reactions. Recent studies also showed that the transition-metal ions (Ti^3+^ and Ti^4+^) present on the LTO surface violently react with the electrolyte, causing the electrolyte solvent to decompose during electrochemical cycling, thereby deteriorating the long-term stability of electrodes made of LTO. These reactions are particularly accelerated in high-temperature environments, which has adverse effects on the cyclability of the electrode [24,25,26]. For LTO to be employed in EV and ESS applications as fast-chargeable anodes, the safety of the LTO material under high-temperature conditions must be ensured; otherwise the accelerated side reactions could result in unexpected explosive accidents. The stability of LTO electrodes has been enhanced by using carbon-based materials to separate LTO from the surrounding electrolyte, resulting in visibly reduced side reactions [27,28]. However, these approaches were not quite effective to suppress gas evolution during electrode cycling, especially at elevated temperatures. Considering that EVs tend to run in the daytime, the electrochemical performances of LTO electrodes should be guaranteed even under certain extreme circumstances.

Herein, aluminum oxide (Al_2_O_3_) was directly deposited on an LTO electrode by atomic layer deposition (ALD), and its cycle-life performance was evaluated at 60 °C to identify an effective way to prevent gas evolution in LTO. The formation of an ultra-thin Al_2_O_3_ layer (~2 nm thickness) on an electrode does not significantly affect the electrical conductivity, and therefore, 98% of the theoretical capacity of LTO is retained even after 500 cycles at 60 °C. LTO without an Al_2_O_3_ layer degrades upon cycling, and it is clearly seen that pouch-type cells made of uncoated LTO inflate as a result of gas generation (CO, CO_2_, H_2_, etc.). The role of the Al_2_O_3_ layer was verified by transmission electron microscopy (TEM) and X-ray photoelectron spectroscopy (XPS). Neither a distortion of the LTO (111) plane nor a change in the oxidation state of Ti was detected on the Al_2_O_3_-coated LTO, whereas a change in the interfacial phase of the uncoated LTO and shifts in the oxidation state (Ti^3+^) were observed after cycling.

## 2. Experimental

### 2.1. Preparation of the LTO Powder and Al_2_O_3_-Coated LTO Electrode

To synthesize LTO powder, Li_2_CO_3_ and TiO_2_ were mixed at a molar ratio of 4.2:5 in acetone and dried at room temperature for approximately one hour to form a Li_2_CO_3_/TiO_2_ mixture. The Li_2_CO_3_/TiO_2_ mixture was heat-treated in a tube furnace in an Ar/H_2_ atmosphere at 850 °C for 10 h, and the resulting powder was further ground to obtain LTO powder [29]. The prepared LTO was uniformly mixed with carbon black and PVDF at a mass ratio of 80:10:10 in *N*-methyle-2-pyrroledine (NMP) to prepare an electrode slurry. The LTO electrode active material slurry was coated to a thickness of ~20 µm onto a Cu foil using a doctor blade, and further vacuum-dried at 110 °C to fabricate a LTO electrode. An Al_2_O_3_-protective layer was deposited with a thickness of several nanometers on the fabricated LTO electrode by ALD. ALD is the most effective approach compared to other deposition methods (such as sol-gel and CVD), since it allows one to adjust the thickness in Å units and uniformly deposit a protective layer [30,31,32,33,34].

### 2.2. Physical Characterization

X-ray diffraction analysis (XRD, Empyrean PAN analytical, Almelo, The Netherlands) was conducted in the range of 2θ = 10°–70° with Cu K α radiation for structural analysis of the synthesized LTO powder. The shape, particle size, and surface changes were observed by field emission-scanning electron microscopy (FE-SEM, JSM-7800F, JEOL, Tokyo, Japan) and transmission electron microscopy (TEM, Tecnai F20 G^2^ Microscope (FEI, Hillsboro, OR, USA) operated at 200 kV). The oxidation state of Ti was investigated by X-ray photoelectron spectroscopy (XPS, AXIS-HIS: KRATOS, Manchester, UK) with incident photon of 1254 eV.

### 2.3. Electrochemical Tests

A pouch-type LTO half-cell was fabricated to analyze the electrochemical characteristics of the materials. The uncoated and Al_2_O_3_-coated LTO were used as the working electrode with an area of 4 cm^2^, and lithium metal was used as the counter electrode. An olefin-based membrane (Celgard 2400, Celgard Inc., Charlotte, NC, USA) was used as a separator. The membrane was inserted into an Al pouch, a 1 M LiPF_6_ electrolyte in a solution of ethylene carbonate (EC) and diethyl carbonate (DEC) (soulbrain, Seongnam, Korea) at a 1:1 volume ratio was also introduced, and the pouch was vacuum-sealed. The electrochemical charge-discharge behaviors and the cycle life performance of the fabricated LTO pouch cell were studied using a galvanostatic charge/discharge cycler (WBCS3000, Wonatech, Seoul, Korea) at 60 °C in a constant-temperature chamber. The voltage range was set between 0.9 V and 2.6 V. The value of 1 C was considered based upon the theoretical capacity of LTO (175 mAh/g). Electrochemical impedance spectroscopy (EIS) of the electrode was conducted using an electrochemical workstation (ZIVE MP1, Wonatech, Seoul, Korea) in a frequency range of 10^5^–10^−1^ Hz.

## 3. Results and Discussion

### 3.1. Synthesis of LTO

**Figure 1:**
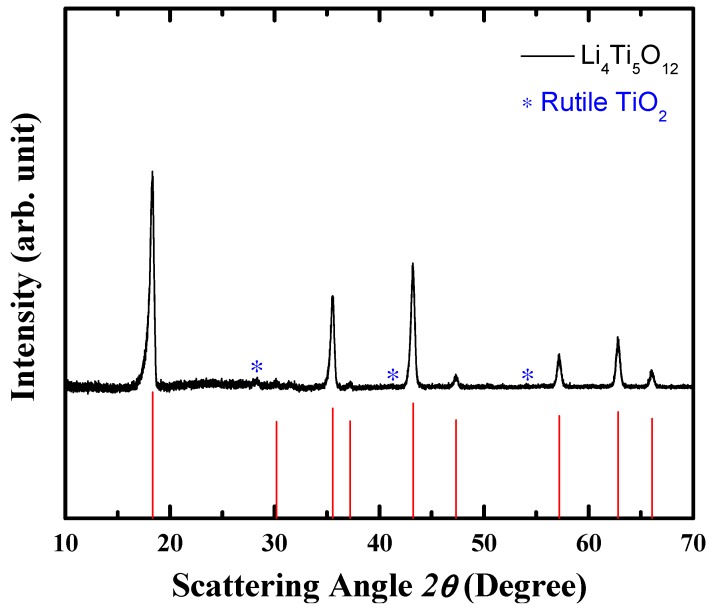
XRD patterns of LTO (Li_4_Ti_5_O_12_) powder. The ideal peak positions and intensities for LTO (JCPDS No. 49-0207) are marked at the bottom. Possible TiO_2_ impurities are denoted by stars.

**Figure 2:**
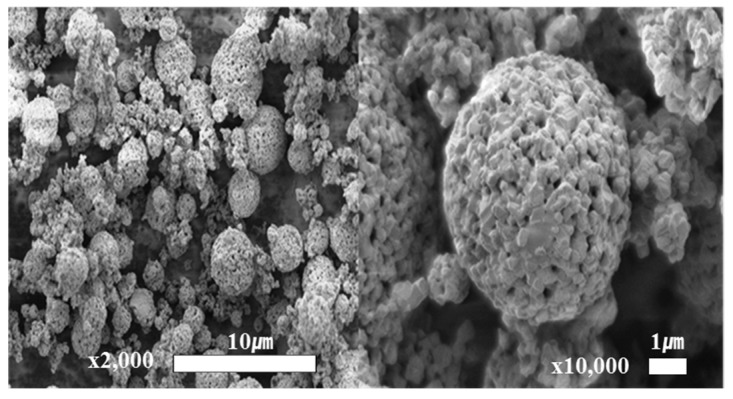
SEM images of synthesized LTO powder.

Figure 1 shows the XRD pattern of the synthesized LTO powder. The diffraction peaks are indexed to the standard diffraction peaks of spinel LTO (JCDPS No. 49-0207). The synthesized LTO powder reveals the spinel structure with little amount of TiO_2_ (rutile) impurities. L. J. Wan et al., claimed that such a small fraction of rutile TiO_2_ at the surface of LTO is beneficial for its rate capability [35]. Furthermore, the shape of the synthesized LTO powder was observed by SEM. As shown in Figure 2, the images clearly display aggregated forms of the LTO, composed of spherical primary particles of several hundreds of nanometers to several micrometers in diameter. The hierarchy of the particles are advantageous for the Li-ion diffusion due to short diffusion path through the nanometer-sized primary particles and high accessibility of electrolyte to the active electrode material owing to the porous structure. The aggregated form of LTO is also beneficial for achieving high tap density of electrodes.

### 3.2. Schematic Diagram and Morphology of Uncoated and Al_2_O_3_-Coated LTO Electrodes

**Figure 3:**
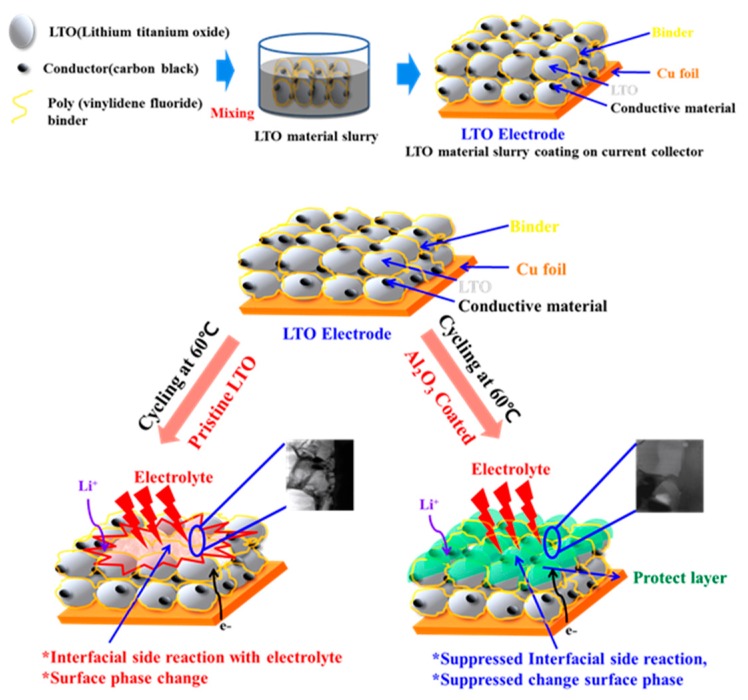
Schematic diagram of (**a**) fabrication of LTO electrode and (**b**) expected phenomena of uncoated and Al_2_O_3_-coated LTO electrode cycled at 60 °C.

**Figure 4:**
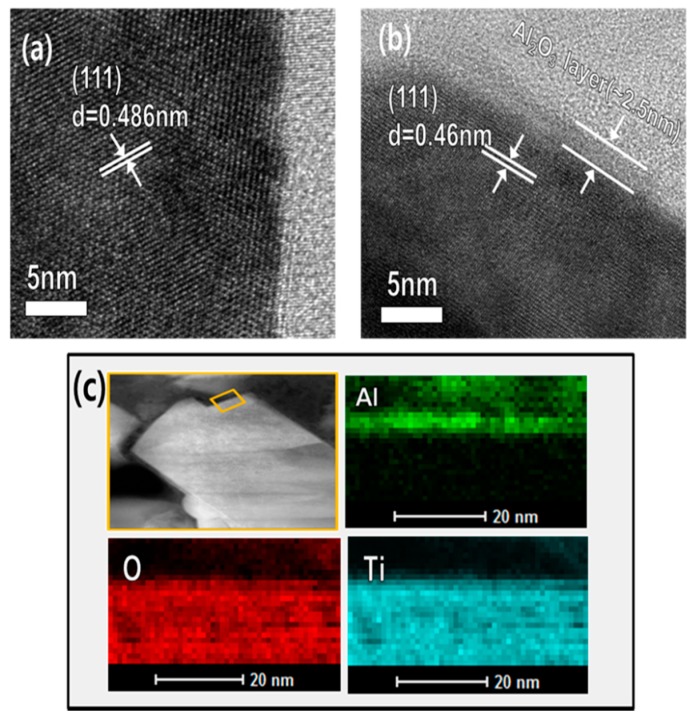
HRTEM Images of the (**a**) uncoated and (**b**) Al_2_O_3_-coated LTO electrode at the pristine state. (**c**) EDX elemental mapping of the Al_2_O_3_-coated LTO electrode.

Figure 3 illustrates the process and effect of the Al_2_O_3_ coating on the electrochemical performance of LTO. As shown in Figure 3a, the electrode was initially constructed with LTO as an active material prior to Al_2_O_3_ coating. During the electrochemical charge/discharge cycles at a temperature of 60 °C, the uncoated LTO electrode surface was directly exposed to the electrolyte, resulting in several side reactions, as a result of which leads to gas (CO, CO_2_, and O_2_) evolution as well as a phase transformation of the crystalline LTO. However, when a thin and conformal Al_2_O_3_ layer was deposited on the LTO electrode by ALD, direct contact with the electrolyte was prevented, resulting in negligible side reactions between the electrolyte and the electrode surface. Thus, the initial state of the LTO electrode was maintained without any damage or phase transformation during the high-temperature electrochemical charge/discharge cycles. The effect of Al_2_O_3_ coating is more notable by a focused-ion beam (FIB) image (Figure S1) after charge-discharge at 60 °C, as shown in Figure 3b. It can also be observed by TEM images that the surface of the LTO electrode was coated by a thin Al_2_O_3_ protective layer. Figure 4a shows the (111) plane of the uncoated LTO electrode surface, confirming that the surface has a typical spinel structure. From Figure 4b,c, it is seen that an Al_2_O_3_ layer is uniformly formed on the surface of the LTO electrode with a thickness of ~2.5 nm. The formation of the protective layer on the electrode surface was carefully confirmed by EDS mapping.

### 3.3. Electrochemical Properties of the Uncoated and Al_2_O_3_-Coated LTO after Cycles at a 60 °C

**Figure 5:**
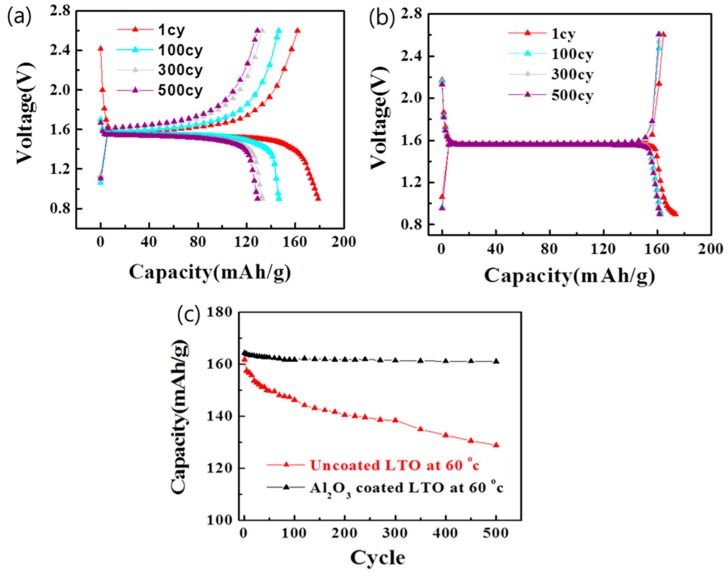
Voltage profiles of the (**a**) Al_2_O_3_-coated and (**b**) uncoated LTO at 60 °C. (**c**) Cycle-life performances of Al_2_O_3_-coated and uncoated LTO at 60 °C. The cells are discharged and charged within a voltage range of 2.6 and 0.9 V at 2 C (2 C = 250 mA/g based upon the theoretical capacity of LTO: 175 mAh/g).

Pouch cells (half cells) were assembled using the uncoated and Al_2_O_3_-coated LTO electrodes to identify and compare the electrochemical charge-discharge behavior and cyclability. For the uncoated one, a relatively significant reduction in capacity with respect to the initial capacity (from 161 mAh/g to 128 mAh/g) was observed after 500 electrochemical charge/discharge cycles at high temperature (60 °C), which demonstrates more unstable behavior (~79% capacity retention), compared to that at room temperature (Figure S2). This observation indicates that the characteristics of the LTO electrode do not change significantly during the electrochemical charge/discharge cycles at room temperature. However, as mentioned in the Introduction, side reactions with the electrolyte are accelerated at high temperatures, which could lead to a reduction in electrochemical stability. To address this problem, the surface of the LTO electrode was coated with Al_2_O_3_, and Figure 5 shows the evaluation and comparison of the characteristics of the Al_2_O_3_-coated LTO. The Al_2_O_3_-coated LTO electrode (Figure 5a) exhibits a remarkably stable charge/discharge behavior as well as minimal reduction in capacity with respect to the initial capacity (from 164 mAh/g to 161 mAh/g) (Figure 5b) with the capacity retained up to ~98% of the initial one. As shown in Figure 5c, the capacity change of Al_2_O_3_-coated LTO during cycles exhibits a behavior that is clearly different than that of the uncoated LTO at high temperature (60 °C). Obviously, the Al_2_O_3_ on the LTO electrode surface separates the electrode surface from the electrolyte during the electrochemical charge/discharge cycles. Moreover, the coated Al_2_O_3_ acts as a protective layer that prevents electrolyte decomposition caused by side reactions between the electrolyte and electrode surface, especially at high temperatures. The Al_2_O_3_ coating improves the electrochemical cycle performance and stability. Meanwhile, the formation of ultra-thin Al_2_O_3_ layer (~2 nm thickness) on LTO electrodes does not significantly affect the electrical conductivity and battery performance, as confirmed by EIS (Figure S3), charge/discharge capacities at various C rates (Figure S4), and cycle-life performance with various thickness of Al_2_O_3_ (Figure S5).

**Figure 6:**
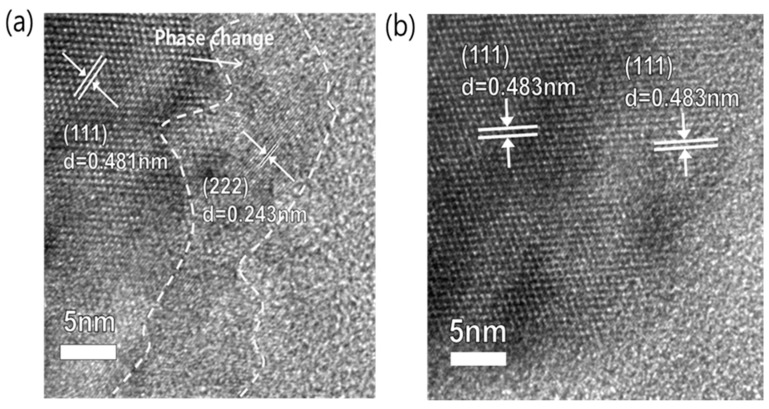
HRTEM images of the (**a**) uncoated and (**b**) Al_2_O_3_-coated LTO electrode after 500 cycles at 60 °C. The electrodes are in a charged state.

**Figure 7:**
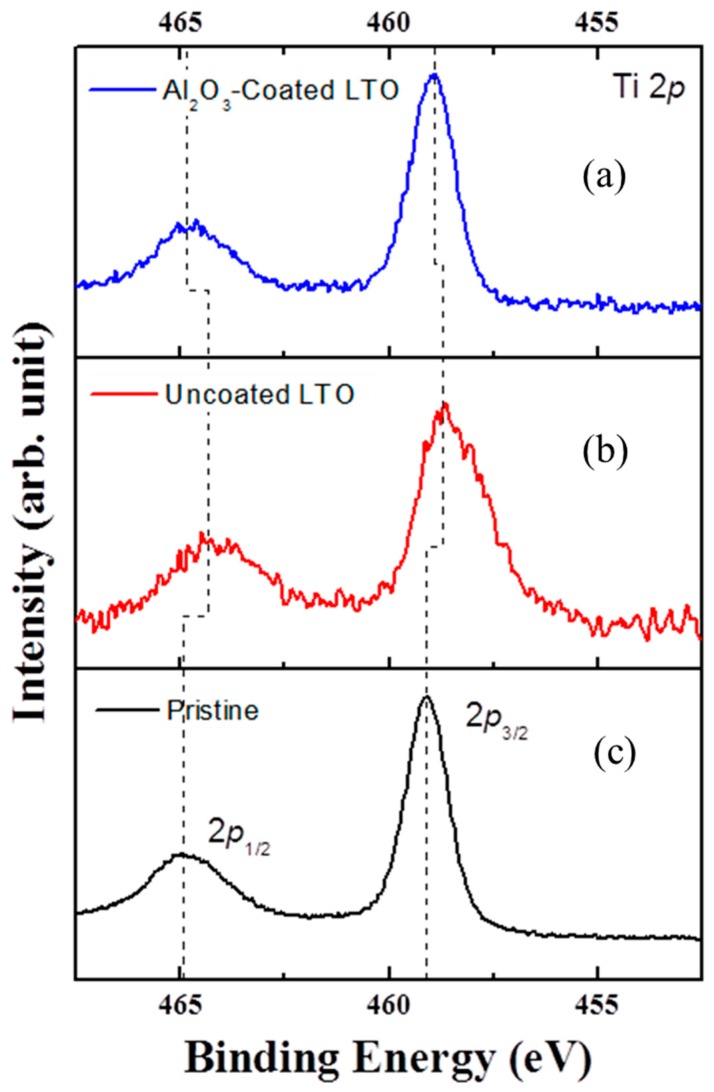
Ti 2*p* X-ray photoelectron spectra (XPS) of the LTO electrodes. (**a**) Pristine state, and charged states of the (**b**) uncoated and (**c**) Al_2_O_3_-coated LTO electrode after 500 cycles. The dashed lines are drawn to guide eyes.

Figure 6 shows the morphological changes in the uncoated and Al_2_O_3_-coated LTO after electrochemical charge/discharge at high temperature (60 °C). As shown in Figure 6a, the (222) phase plane, which is different from the (111) phase plane with a typical spinel structure, was identified on the outermost layer of the uncoated LTO electrode surface. At the high temperature of 60 °C, charging the uncoated LTO electrode accelerates the side reaction between the Ti^3+^ ions remaining on the electrode surface and the electrolyte solvent, which results in the decomposition of the electrolyte and the generation of gases. Furthermore, this side reaction depletes O_2_^2−^ ions from the outermost electrode, leading to partial phase change of LTO crystals, as seen in Figure 6a. The side reaction is reflected as a reduction in the electrochemical capacity upon cycling, which would be more serious in applications for large-sized batteries [24,29]. However, the Al_2_O_3_-coated LTO exhibited no sign of phase change on the surface of the electrode, maintaining the (111) phase plane with a spinel structure even after 500 cycles at 60 °C (Figure 6b). The Ti 2*p* X-ray photoelectron spectra (XPS) of the uncoated and Al_2_O_3_-coated LTO electrodes (Figure 7) show that Ti 2*p* spectrum of the uncoated LTO shifts to lower binding energy compared to the pristine and Al_2_O_3_-coated LTO, indicating that depleted O^2−^ ions accompanies the permanent change of Ti oxidation state, which, otherwise, should have turned back to nearly Ti^4+^ state when the electrode is charged. The FIB images also corroborate that the Al_2_O_3_-coated LTO electrode maintained a stable state without damages or changes on the electrode level, compared to that of the uncoated LTO (Figure S1).

## 4. Conclusions

In order to improve the high-temperature stability of the LTO (Li_4_Ti_5_O_12_) anode, an ultra-thin and uniform Al_2_O_3_ protective layer (~2 nm thickness) was directly coated on the electrode surface using ALD. The Al_2_O_3_-coated LTO electrode showed an excellent electrochemical cycle-life performance, retaining 98% of the initial capacity after 500 cycles. The outermost (111) plane of the coated LTO was maintained without any damage nor phase transformation, compared to the uncoated LTO electrode. Al_2_O_3_ acted as a protective layer that suppressed side reactions at the interface between the LTO electrode and electrolyte, which could have been accelerated at high temperature. Accordingly, the formation of an ultra-thin and uniform Al_2_O_3_ protective layer onto the LTO electrode surface is an effective method to improve the high-temperature stability of LTO electrodes.

## Supplementary Materials

The following are available online at http://www.mdpi.com/1996-1944/11/5/803/s1, Figure S1: FIB images the (**a**) uncoated and (**b**) Al_2_O_3_-coated LTO after 500 cycle at 60 °C. The electrode are in a charged state. Figure S2: Charge and discharge curves of uncoated LTO (**a**) at 25 °C, (**b**) at 60 °C, (**c**) Cycle-life performances of the uncoated LTO cycled at 25 °C and 60 °C. The cells are discharged and charged within a voltage range of 2.6 and 0.9 V at 2 C. (2C = 250 mA/g based upon the theoretical capacity of LTO: 175 mAh/g). Figure S3: Nyquist plots of the uncoated and Al_2_O_3_-coated LTO electrodes. Figure S4: Galvanostatic charge-discharge tests of the uncoated and Al_2_O_3_-coated LTO electrode at different current densities varied from 0.1 to 20 C. Figure S5: Comparison of the Al_2_O_3_-coated LTO electrodes having different thicknesses.
