# Supplementary material for: Highly-Stable Li4Ti5O12 Anodes Obtained by Atomic-Layer-Deposited Al2O3
Source: Materials (Basel). 2018 May 16;11(5):803. doi: 10.3390/ma11050803 (PMC5978180; doi:10.3390/ma11050803)
Supplement: Supplementary file 1 [file materials-11-00803-s001.pdf]

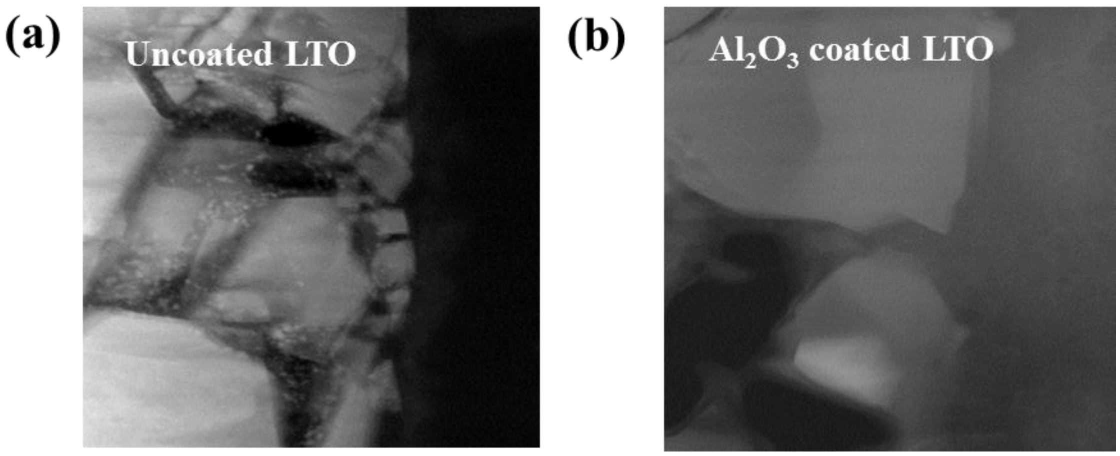

Figure S1. FIB images the (**a**) uncoated (**b**) Al_2O_3-coated LTO after 500 cycle at 60℃. The electrodes are in a charged state.

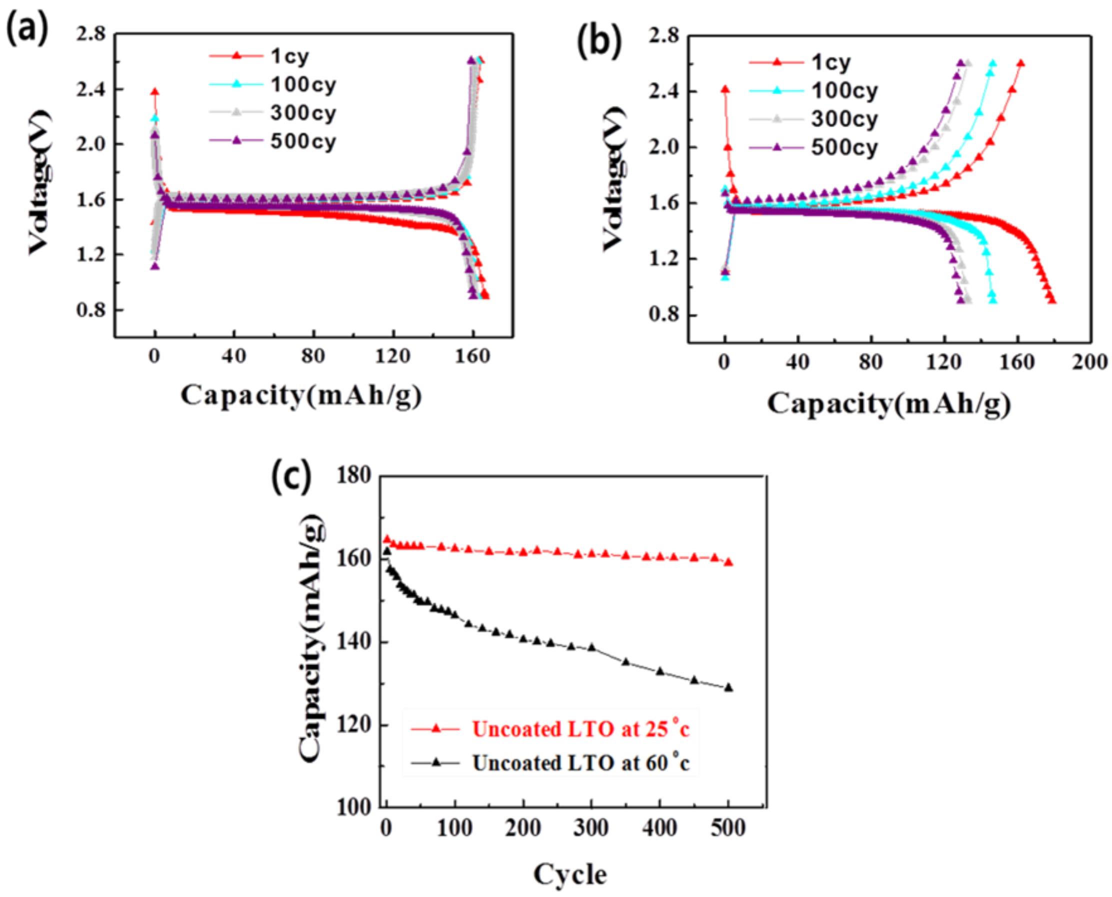

Figure S2. Charge and discharge curves of uncoated LTO (**a**) at 25˚C and (**b**) at 60˚C. (**c**) Cycle-life performances of the uncoated LTO cycled at 25˚C and 60˚C. The cells are discharged and charged within a voltage range of 2.6 and 0.9 V at 2 C. (2 C = 250 mA/g based upon the theoretical capacity of LTO: 175 mAh/g).

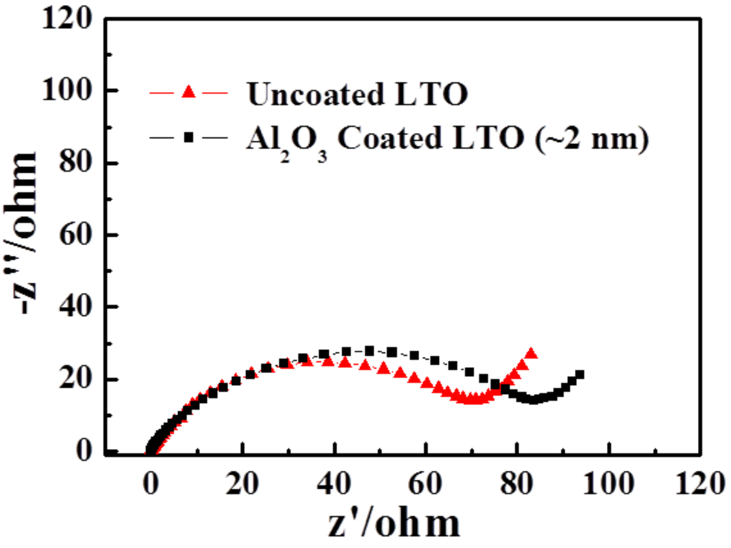

Figure S3. Nyquist plots of the uncoated and Al_2O_3-coated LTO electrodes.

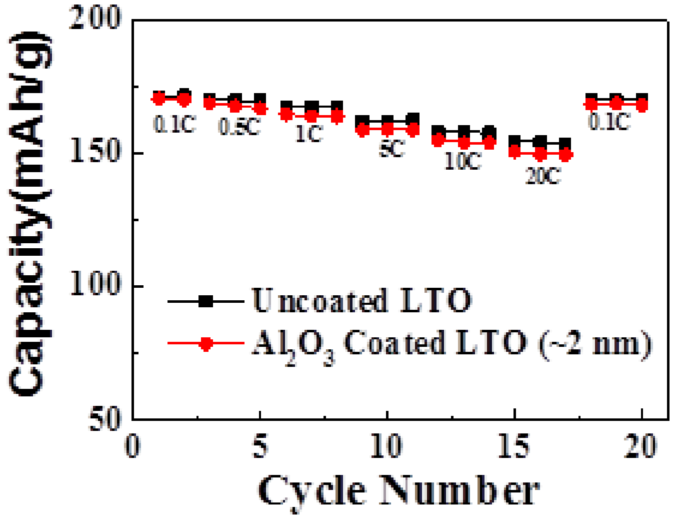

Figure S4. Galvanostatic charge-discharge tests of the uncoated and Al_2O_3-coated LTO electrode at different current densities varied from 0.1 to 20 C.

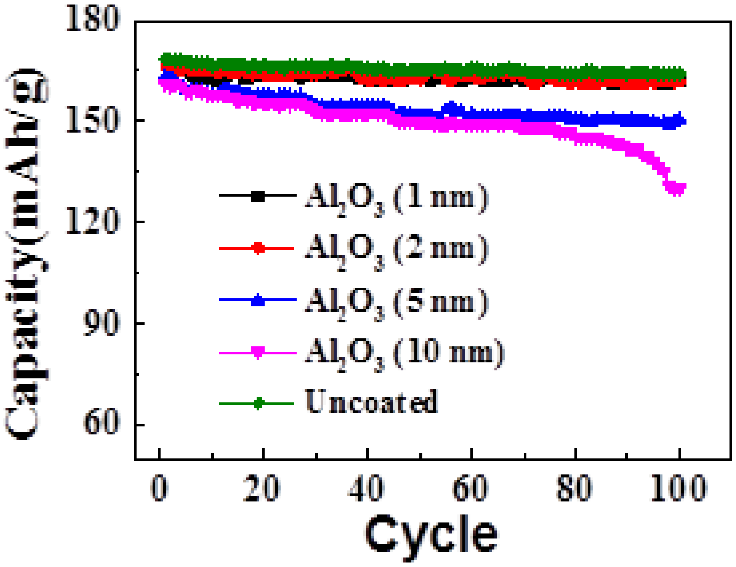

Figure S5. Comparison of the Al_2O_3-coated LTO electrodes having different thicknesses.
